# Conceptual barriers to palliative sedation: insights from focus group interviews with specialist palliative care professionals

**DOI:** 10.1186/s12904-025-01931-y

**Published:** 2025-12-02

**Authors:** Solveig Torvund, Lisbeth Thoresen, Morten Magelssen, Olav Magnus Fredheim

**Affiliations:** 1 Department of Palliative Medicine, Division of Surgery, Akershus University Hospital, Lørenskog, Norway; 2 Faculty of Medicine, Institute of Clinical Medicine, University of Oslo, Oslo, Norway; 3 Faculty of Medicine, Institute of Health and Society, University of Oslo, Oslo, Norway

**Keywords:** Palliative sedation, Symptom management, Euthanasia, Sedatives, Palliative care, Intention, Qualitative research, Conceptual analysis, End-of-life care

## Abstract

**Background:**

Palliative sedation is considered an essential measure when other methods cannot provide sufficient symptom control in end-of-life. Prior research suggests that health care professionals find boundaries between palliative sedation, routine symptom management and euthanasia ambiguous. Appropriate implementation of palliative sedation depends on palliative care clinicians being confident in conceptual understanding of palliative sedation. The aim of this study was to understand specialist palliative care professionals' conceptualization of distinctions between symptom management, palliative sedation, and euthanasia; how perceived boundary ambiguities act as barriers to timely initiation of palliative sedation; and potential strategies to overcome these barriers.

**Methods:**

This is an explorative qualitative phenomenological study, based on focus group interviews, utilizing reflexive thematic analysis. Four focus groups were interviewed at specialist palliative care departments across four Norwegian hospitals. Participants were 11 nurses and 12 physicians with at least one year of experience within specialist palliative care.

**Results:**

Through thematic analysis two core themes were developed; the distinction between symptom management and palliative sedation, and the distinction between palliative sedation and euthanasia. Important sub-themes were definitional uncertainties, complexity of gradual medication titration, timing of sedation, indications for sedation, communication, and concerns about hastening death.

**Conclusion:**

Even within specialist palliative care, different end-of-life treatment concepts are not experienced clear-cut. These conceptual ambiguities impede appropriate initiation of palliative sedation. Overcoming such barriers requires multi-level interventions: enhancing practitioners' definitional knowledge, fostering awareness of clinical intention during end-of-life treatment, promoting interprofessional dialogue, and developing more explicit clinical guidelines.

**Supplementary Information:**

The online version contains supplementary material available at 10.1186/s12904-025-01931-y.

## Background

Palliative sedation for terminally ill patients involves pharmacologically reducing consciousness to alleviate intractable suffering at the end of life [1]. A condition is considered refractory when no existing methods can provide sufficient relief within an acceptable timeframe or without imposing unacceptable negative consequences for the patient. It is a well-recognized challenge that diverse terminology of palliative sedation causes inconsistency in study populations, case definitions and discussions about clinical practice [2]. While the term “deep and continuous palliative sedation” frequently appears in scientific literature, the European Association for Palliative Care’s framework from 2024 emphasizes proportionality and focus on sedation deep enough to achieve the primary objective of symptom relief [1]. In this paper, “palliative sedation” will be used interchangeably with proportional sedation and deep and continuous palliative sedation.

Sedatives have a central role in symptom management at the end of life, as treatment for anxiety, agitation and delirium [3–5]. Some of the same drugs are also used for euthanasia and physician assisted suicide [6]. Still, it has been strongly argued that the difference between palliative sedation and euthanasia is clear-cut, with fundamental differences in both the intention, procedure and the desired outcome [7–10].

A recent survey among Norwegian pulmonologists revealed uncertainty regarding both definitions, the difference between palliative sedation and euthanasia, and interpretation of guidelines [11]. Prior research suggests that also experienced palliative care clinicians report that distinctions between symptom management, palliative sedation, and euthanasia may be ambiguous [12]. It appears that clinicians might focus more on superficial similarities in outcomes than differences in procedure and intention [13]. This is, however, balanced with reports of clinicians viewing palliative sedation as an alternative to euthanasia and actively educating others about this [14]. For appropriate and timely initiation of palliative sedation it is crucial that clinicians feel confident in their conceptual understanding of end-of-life treatments [15].

## Methods

### Aim

Our initial objective was to explore healthcare professionals’ experiences with decision-making processes regarding palliative sedation through focus group interviews. However, data analysis revealed significant conceptual uncertainty in the use and understanding of terminology. Upon recognizing the substantive nature of these ambiguities, we reoriented our analytical approach with a refined research question. Consequently, we seek to understand how specialist palliative care physicians in Norwegian clinical practice experience and conceptualize boundaries between symptom management, palliative sedation and euthanasia, with the latter being illegal in Norway. Our focus centers on identifying the factors contributing to perceptions of such uncertainties, exploring if conceptual unclarity itself can impact decision-making processes.

### Choice of method

The method of focus group interviews was chosen because of its ability to provide unique access to social norms, enabling participants to build upon, challenge, clarify, and nuance each other’s perspectives, thereby generating richer, more complex data [16]. The approach is based on a phenomenological tradition as the study has an exploratory aim [17], whilst elements from a more descriptive design also have been integrated throughout the process.

### Setting

The Norwegian healthcare system is publicly funded and structured across two levels: the primary care with family practitioners and community care services, and specialist care with hospitals and specialist services. Hospitals provide specialist palliative care, organized through ambulatory teams or dedicated hospital wards [18]. Palliative sedation is administered to hospitalized patients or by specialist palliative care services. Consequently, the focus group interviews were conducted within specialist palliative care settings. Two interviews took place in palliative care hospital units and two in palliative teams sited at hospitals. The staff numbers ranged from 8 to 50 nurses and physicians.

### Interviewees

Through purposive sampling, we included 11 nurses and 12 physicians with a minimum of one year of experience in specialist palliative care, seeking a high level of information power [19]. By ensuring variation in participants’ workplaces and experiences we aimed to mitigate the potential for findings to be overly influenced by local institutional culture. Incorporating different professions within the same focus groups aligns with the multidisciplinary nature of clinical work in palliative medicine.

The participants had an average of 10.9 years’ experience in specialist palliative care. The age ranged from 27 to 68 years, with an average of 54 years. 4 nurses and 6 physicians worked in hospital based palliative teams, while 7 nurses and 6 physicians had specialist palliative hospital wards as their clinical setting Table 1.

**Table 1:** Overview of themes developed through analysis

Core themes	Sub themes
The distinction between symptom management and palliative sedation	Definitional uncertainty Indication uncertainty Gradual medication titration Timing
The distinction between palliative sedation and euthanasia	Definitional uncertainty Fear of hastening death Communication

### Research team and reflexivity

All four researchers involved in this project possess healthcare professional backgrounds, with two of them holding senior positions as a department head (OMF) and a senior consultant (ST) in a palliative care unit, respectively. Consequently, the researchers’ professional pre-understanding and disciplinary perspectives will have influenced the development of the interview guide, the conduct of interviews, and analysis of the interview material. Given the relatively limited size of the palliative care professional community in Norway, it was also inevitable that some participants and researchers would have prior professional acquaintance. Throughout the research process, we maintained a consistent awareness of how these factors influenced our analysis, and we actively engaged in critical reflection regarding its implications for our interpretations.

### Data production

The interviews were conducted between June and October 2024. Participants from the same hospital were interviewed in a single group at their workplace, resulting in four group interviews. Prior to recording, participants completed a sociodemographic questionnaire and signed informed consent forms.

The interviews were facilitated by ST, and in the two first interviews, experienced qualitative researchers (LT and MM, respectively) participated as co-moderators and mentors. Co-moderators initially assumed an observational role and took notes, but in the latter part of the interviews they intervened, requesting further information on topics that had not been sufficiently explored. We used a semi-structured interview guide developed by the research team. All interviews were audio recorded and transcribed verbatim. The average interview duration was 68 min (ranging from 58 to 78 min).

The interviews centered on participants’ authentic clinical experiences with palliative sedation. Participants were asked to share their perspectives on the concept itself and its associated terminology. Further, they were asked to describe experiences with cases where palliative sedation had been carried out, describe situations where sedation was not administered but could have been appropriate, explain the decision-making process leading to palliative sedation, and discuss challenges encountered.

Literature describes focus group interviews to differ significantly from individual interviews by prioritizing the interaction between participants over individual statements [20, 21]. Such interaction was notably present in our interviews, as participants throughout all interviews built upon each other’s comments continued associating ideas. New insight was clearly developed as the conversations progressed, evident in statements such as ‘I haven’t actually thought about this before, but I now see that.‘. The clinical experience with palliative sedation varied within the groups, and at two of the four locations, palliative sedation was not a well-established practice. Nevertheless, all participants engaged actively throughout the interviews. We observed minimal hierarchy and that both physicians and nurses participated equally in the discussions, with limited interfering from moderators. We also observed that participants spoke freely and did not appear reluctant to express themselves explicitly, daring to make pointed statements or share vulnerable experiences.

### Analysis

We employed thematic analysis [16], a method valuable for investigating under-researched areas, as it recognizes how individuals construct meaning from their experiences and how broader social contexts influence these interpretations. Our analysis exhibits a reflexive character [22], which permits the researchers to maintain an active and positional role, wherein subjectivity is utilized as a resource, contingent upon the exercise of reflexivity. Furthermore, the method is characterized by being an evolving process of noticing relevant meaning in a dataset, labeling them with codes, and ultimately clustering these codes to formulate themes [23]. Three members of the research group independently reviewed the transcripts and documented analytical decisions with transparency. To ensure data reporting quality, we applied the Standards for Reporting Qualitative Research (SRQR) guideline [24], Reflexive thematic analysis reporting guidelines (RTARG) [25] and Consolidated Criteria for Reporting Qualitative Research (COREQ) [26].

### Data protection and research ethics

Participation was based on informed consent, with data security and anonymity managed in accordance with data protection regulations. The project was approved by the Data Protection Officer at Akershus University Hospital. Based on a remit assessment form the Regional Committee for Medical and Health Research Ethics South-Eastern Norway evaluated the project as health services research not requiring ethical approval (reference number 687379). Data were stored in a secure server environment accessible solely to the researchers. The project was conducted in accordance with the principles of the Helsinki declaration [27].

## Results

Experience with palliative sedation varied widely among the participants. Some reported to regularly consider and utilize it, while others had rarely encountered it, and even questioned its utility in their practice. Nevertheless, consensus existed that palliative sedation could be beneficial in situations involving short life expectancy and refractory suffering. Initially, participants reported confidence regarding the definition of palliative sedation, and distinctions from other end-of-life approaches. However, during interviews, scenarios emerged where these distinctions proved difficult. Two areas appeared particularly ambiguous: the distinction between routine use of sedatives for symptom relief versus palliative sedation, and between palliative sedation and euthanasia. In this paper, we explore these themes and discuss how uncertainty around boundaries and definitions can represent significant professional and ethical barriers.

### Core theme 1: the distinction between symptom management and palliative sedation

#### Definitional uncertainty

Participants provided examples illustrating how sedation terminology can be applied inconsistently across different clinical settings. They noted that definitions could vary substantially not only among palliative care professionals but also among healthcare workers in non-palliative departments and community healthcare environments. The multiple definitions in medical literature were further identified as a source of confusion.

*I find these terms really tricky. I mean… you could argue that palliative sedation is also just symptom management. That’s all it really is. You’re relieving pain*,* but not with pain medication*,* instead with sedatives. And when we think about restlessness and anxiety… that anxiety we treat with Midazolam… isn’t that kind of sedation*,* right? I find it all a bit… challenging. (Physician 3)*

*And there have been quite a few nurses who’ve mixed up palliative sedation with the standard “medication toolkit”. They’d hear about palliative sedation and think it was just those “four final medications”. That’s created some confusion. It’s become a bit of a topic of discussion on the ward. (Nurse 8)*

*In quite a few papers in the literature*,* they actually call it palliative sedation – what we call symptom management. They use “sedation” even for smaller doses. (Physician 5)*

#### Indication uncertainty

Discussions across all interviews explored various indications for initiating palliative sedation. While most participants associated palliative sedation primarily with cancer-related pain or respiratory failure, some reported cases dominated by existential suffering. However, some stated that psychological symptoms should ideally be addressed through routine palliative treatment. Even in late-stage life circumstances, palliative sedation was by some not recommended when psychological distress was the sole presenting symptom. Some even expressed uncertainty regarding whether palliative sedation for psychological symptoms was a legally and ethically permissible treatment at all. Collectively, the participants’ experiences underscored the particular challenge of differentiating between routine symptom management and what should be defined as palliative sedation in cases where psychological suffering appears to dominate.

*The thing with defining palliative sedation is that you’re supposed to be really cautious about using it for psychological symptoms. It should primarily be for physical symptoms*,* you know? But of course*,* the physical and psychological distress of a patient experiencing respiratory failure*,* for instance*,* are so deeply interconnected… (Physician 12)*.

*You could kind of problematize this a bit… like*,* is it sometimes… might it become a shortcut out of dealing with the actual problems? (Physician 2)*

#### Gradual medication titration

Participants provided numerous examples where sedatives titrated for anxiety or symptom management ultimately resulted in complete sedation. Some participants told of experiencing professional and ethical discomfort in such circumstances, as these situations could give a sense of procedural dishonesty, particularly when a change of intention from symptom relief to sedation wasn’t openly acknowledged and discussed among staff. Some noted that by conceptualizing the intervention as a gradual escalation of symptomatic treatment rather than formal palliative sedation, they could bypass the complex ethical deliberations inherent in such decision-making.

*I find that it can become very ambiguous*,* and I’ve questioned myself… “Are we pursuing sedation here? Or is this purely anxiety management?” (…) What exactly are our intentions? And when no one discusses it*,* it becomes an unspoken issue. (Nurse 5).*

Conversely, other participants explained that this approach facilitated communication with patients and families. The gradual nature of the intervention with routine symptom treatment allowed families to more readily comprehend and accept the process, making it psychologically more accessible.

*Many patients die under Midazolam treatment*,* unconscious*,* but since the treatment was initiated to reduce anxiety*,* unconsciousness is considered a side effect rather than an intended outcome (…) This allows us to circumvent certain ethical considerations. It’s also more comprehensible for next-of-kin. (Physician 11)*

#### Timing

Participants frequently reported uncertainty regarding whether all alternative interventions had been exhausted and whether the remaining life expectancy was sufficiently brief to justify inducing sedation. In scenarios where participants already struggled to differentiate symptom management with sedation as a side effect and genuine palliative sedation, the decision-making process was perceived as particularly complex. Clinicians described tending to increase doses of palliative medication rather than transitioning to the palliative sedation procedure.

*The patient was receiving high doses of Midazolam and Ketamine*,* and the situation was… suboptimal. Ideally*,* we should have paused and said*,* “We need a team meeting*,* we need to reconsider our approach*,*” but instead I said “Let’s try increasing the Ketamine*,* add some Nozinan” to bridge until the next day… (Physician 5)*.

### Core theme 2: the distinction between palliative sedation and euthanasia

#### Definitional uncertainty

Despite participants expressing confidence in distinguishing between performing euthanasia and palliative sedation, they provided numerous examples from their clinical practice where such differentiation had been experienced as challenging. Some participants recounted personal experiences of ethical discomfort or uncertainty, while many shared narratives of similar emotional responses among their colleagues or patients’ families. Some described being questioned about whether their actions constituted euthanasia, causing significant discomfort. As a result, many participants called for clearer guidelines.

*The patient was a young woman who was about to be separated from her children and husband*,* and we were going to… assist in her death*,* in a way. I found that very challenging at the time. (Nurse 2)*

*I’ve heard colleagues say*,* “They’re basically doing euthanasia here!” I didn’t really know enough about palliative sedation*,* and I didn’t see it that way either*,* because the patient was getting Midazolam and Ketamine (…) But I really felt it*,* you know*,* I found it really uncomfortable*,* especially since I was the only senior doctor there at that time. I felt vulnerable. (Physician 4)*

*“There have been cases in palliative care where people have had to face the question of euthanasia legally… and I find it difficult that guidelines on this are so unclear”. (Physician 7)*

#### Fear of hastening death

Many participants described an underlying apprehension that palliative sedation might inadvertently precipitate death, thereby potentially blurring the boundaries between this intervention and euthanasia. This concern manifested in various anxieties, particularly regarding the practice of not monitoring the patient, and uncertainties surrounding the management of unforeseen complication, such as loss of airway patency.

*For an anesthesiologist*,* it’s deeply unsettling to sedate someone without full monitoring of oxygen saturation*,* blood pressure*,* and pulse… It’s an uncomfortable position to be in. It’s like: “We don’t need blood tests here*,*” or “We don’t need blood pressure measurements here”*,* you know? “We just need to ensure the patient is lying still in bed and appears comfortable.” (Physician 8)*.

#### Communication

The importance of an open dialogue about palliative sedation as a potential intervention during communication with patients and their families was repeatedly emphasized throughout the interviews. This was deemed crucial to prevent conceptual confusion and to enhance patient and family understanding of the distinction between palliative sedation, other symptom management, and euthanasia.

*I have encountered next-of-kin who were confused about the concepts*,* but after thorough dialogue with the doctors*,* they expressed understanding of the difference. (Nurse 7)*

## Discussion

Though most of our participants reported feeling confident about definitions regarding palliative sedation, they still presented numerous examples from their clinical practice illustrating ambiguous boundary considerations, misunderstandings, and moral discomfort both internally and among other healthcare professionals, patients or next-of-kin. These complexities were particularly evident in the experienced indistinct boundaries between standard symptom management and palliative sedation, and to some extent, between palliative sedation and euthanasia.

Our analysis revealed varied attitudes toward the transition from routine symptom management to formal palliative sedation. Participants noted discrepancies between guidelines and practice, particularly when sedatives for anxiety were gradually increased to levels causing continuous sedation. Some expressed ambivalence about this approach, perceiving it as dishonest or circumventing guidelines. Others found it psychologically beneficial to frame sedation as a side effect rather than the primary intention, facilitating coping mechanisms for patients, families, and healthcare providers. The latter is in line with findings from a German qualitative study [28], where respondents tended to label intentional reduction of consciousness as a wanted side effect, and not as palliative sedation. Further, our findings support previous assumptions that it may be perceived as difficult to distinguish between treatments based on intention when the outcome for the patient is the same [13]. We also find it noteworthy that some participants in this and previous studies [29–31] often characterize sedation as a side effect of anxiolytics, overlooking that reduced consciousness also is frequently part of the natural dying process.

In the publication “The role of palliative sedation” from 2024 [32] contributors aimed to elucidate aspects of palliative sedation, delineating its conceptualization and implementation across European palliative care environments. The publication highlights the persistent challenge of knowledge deficits and underutilization of professional recommendations, despite increasingly explicit guidelines. Our study’s participants echoed these concerns, illuminating conceptual unfamiliarity and misunderstandings encountered among healthcare professionals and patients’ next-of-kin. They expressed concerns that not all collaborating partners are well-acquainted with the Norwegian guidelines, while simultaneously acknowledging their own experiences of perceiving the guidelines as ambiguous. Participants highlighted instances where clinicians hesitated to classify interventions as palliative sedation, driven by underlying uncertainty regarding appropriate indication and timing, and an apprehension that such treatment might be perceived as potentially hastening death. This hesitancy may result in actual cases of palliative sedation being labeled as symptom treatment and possibly lead to violation of guidelines for palliative sedation. Furthermore, this reluctance might also lead to a slow titration of sedatives to the level necessary to achieve sedation. In such cases, the uncertainty represents a barrier to timely and appropriate initiation of palliative sedation.

Even though participants were cognizant that the intention of the treatment differs fundamentally between palliative sedation and euthanasia, they still described an unease about the potential for misinterpretations if a patient dies shortly after initiating sedation. This suggests that, even with a clear theoretical framework, the clinical reality may still present situations where one’s intention may be questioned either by oneself, colleagues or next of kin. Additionally, there could be situations where even the clinicians themselves have ambivalent, uncertain, multiple or varying intentions throughout a decision-making process [33]. An awareness of this, and efforts to make one’s own intentions clear and explicit, are of great importance when facing situations where boundaries might be perceived as unclear [34].

The emergence of very diverse experiences and attitudes, and the experience of blurry demarcations within highly specialized palliative care settings, underscores the inherent complexity of palliative sedation as an intervention. One may argue that there will always be clinical situations so intricate they defy categorization. Nevertheless, one must continue exploring strategies helpful to make the clinical landscape less blurry, and it seems that this process should occur across three different levels: (1) The personal level, ensuring thorough familiarity with concepts, enhancing clinical confidence and awareness of one’s own intentions in various clinical situations; (2) facilitation of dialogue within the specialist community to establish professional consensus and promote a more standardized approach when engaging with the broader healthcare system, patients and families; and (3) development of more explicit local and national guidelines, as also advocated in a recent publication recommending revision of the current Norwegian guidelines [35].

### Strengths and limitations

Qualitative research will always be contextual [36] and when interpreting the material in our study, it is important to bear in mind that the palliative care field is characterized by diverse local conditions, cultural context, and regulatory frameworks. Further, the focus group interview setting may not have provided complete perception of anonymity for the participants, potentially introducing a risk for socially desirable response bias. The risk of such response bias might be particularly relevant in this study due to the reported ambiguity related to the boundaries between palliative sedation and euthanasia. Additionally, hierarchical interpersonal dynamics among colleagues could influence interactions. We ensured that these risks were limited by thorough preparation of the interview guide, by adequate information to the participants before and during the interviews, by making sure that all opinions could be voiced, and by emphasizing anonymization of data. Another strength of the study is the purposive sampling of specialist palliative care professionals, both nurses and physicians, from different hospitals and care settings, believed to provide high information power. Research credibility was further strengthened through independent analysis of the material, which may ensure a richer reading of the data [22] and by the research group maintaining a high level of reflexivity by acknowledging their own preconception and personal relation to the field.

## Conclusion

Optimal and timely initiation of palliative sedation requires practitioners’ conceptual certainty and confidence in their professional community’s collective understanding of the distinctions between routine symptom management, palliative sedation and euthanasia. However, our analysis suggests that specialist palliative care clinicians experience unclear boundaries between these interventions. This can represent significant professional and ethical barriers to timely initiation of palliative sedation when otherwise appropriate. The findings highlight not only a need for guidelines to more clearly address the definitions, but also the necessity of an open dialogue within the palliative care sphere concerning how the boundaries should be understood and communicated among professionals and to patients and their families.

## Supplementary Information

Supplementary Material 1

## Data Availability

Data are stored in a secure server environment accessible solely to the researchers. Sharing of transcripts for verification of findings will depend on approval from the data protection authority (The Data Protection Officer at Akershus University Hospital). Requests for access to the transcripts can be submitted to the corresponding author.
