# Supplementary material for: Conceptual barriers to palliative sedation: insights from focus group interviews with specialist palliative care professionals
Source: BMC Palliat Care. 2025 Dec 2;24:299. doi: 10.1186/s12904-025-01931-y (PMC12673737; doi:10.1186/s12904-025-01931-y)
Supplement: Supplementary file 1 — Supplementary Material 1 [file 12904_2025_1931_MOESM1_ESM.pdf]

Interview Guide for Focus Group Interviews with Healthcare Personnel

BARRIERS TO PALLIATIVE SEDATION

1. Presentation of the project and information about the interview situation
2. Collection of background information according to protocol/information sheet
3. Main section:
    - Participants are asked to share an experience one or more members of the group have had with palliative sedation. The other participants are invited to comment, provide additional information, or ask questions about the situation being described.
    - Participants are asked to share an experience one or more have had where palliative sedation was proposed but not initiated
    - We will ask follow-up questions throughout to elicit information about:
        - Who first raised the topic of palliative sedation in the situation you described?
        - How does one proceed once the topic has been raised? Who is responsible for next steps?
        - How are the patient and family involved/engaged?
        - How are the practical aspects of palliative sedation handled at your workplace? What are the responsibilities and roles of nurses and physicians?
        - Which patients are palliative sedation most relevant for? Are there characteristics that define these patients?
        - What is usually experienced as most challenging before the decision about palliative sedation is made?
        - What is usually experienced as most challenging once palliative sedation has been initiated?
        - How have you perceived the family members' needs and reactions in these situations? What do you discuss with the family members?
        - Do you have local guidelines for palliative sedation? How are these used? Are they perceived as useful? Can there be challenges related to the guidelines?

- Do you have reflection groups/peer debriefing? Is the clinical ethics committee used in any case?

4. Conclusion: Summary and thanks. Information about the possibility of participants contacting the research group afterwards. Information that the research group may also need to contact participants afterwards if something is unclear, provided that the individual participant consents to this.
